# Clinical Relevance of the Microbiome in Odontogenic Abscesses

**DOI:** 10.3390/biology10090916

**Published:** 2021-09-15

**Authors:** Sebastian Böttger, Silke Zechel-Gran, Daniel Schmermund, Philipp Streckbein, Jan-Falco Wilbrand, Michael Knitschke, Jörn Pons-Kühnemann, Torsten Hain, Markus Weigel, Can Imirzalioglu, Hans-Peter Howaldt, Eugen Domann, Sameh Attia

**Affiliations:** 1 Deptartment of Oral and Maxillofacial Surgery, Justus-Liebig-University Giessen, University Hospital Giessen, Klinikstrasse 33, D-35392 Giessen, Germany; Daiel.Schmermund@uniklinikum-giessen.de (D.S.); Philipp.Streckbein@uniklinikum-giessen.de (P.S.); Jan-Falco.Wilbrand@uniklinikum-giessen.de (J.-F.W.); Michael.Knitschke@uniklinikum-giessen.de (M.K.); HP.Howaldt@uniklinikum-giessen.de (H.-P.H.); sameh.attia@dentist.med.uni-giessen.de (S.A.); 2 Institute of Medical Microbiology, Justus-Liebig-University Giessen, D-35392 Giessen, Germany; Silke.Zechel@mikrobio.med.uni-giessen.de (S.Z.-G.); Torsten.Hain@mikrobio.med.uni-giessen.de (T.H.); Markus.Weigel@mikrobio.med.uni-giessen.de (M.W.); Can.Imirzalioglu@mikrobio.med.uni-giessen.de (C.I.); 3 Institute of Medical Informatics, Justus-Liebig-University Giessen, D-35392 Giessen, Germany; Joern.Pons@informatik.med.uni-giessen.de; 4 German Center for Infection Research (DZIF), Justus-Liebig-University Giessen, D-35392 Giessen, Germany; Eugen.Domann@mikrobio.med.uni-giessen.de; 5 Institute of Hygiene and Environmental Medicine, Justus-Liebig-University Giessen, Schubertstrasse 81, D-35392 Giessen, Germany

**Keywords:** oral microbiome, bacteriome, odontogenic abscess, 16S rRNA gene analysis, polymicrobial infection, anaerobic infection, bacterial culture, microbiome analysis

## Abstract

**Simple Summary:**

Odontogenic infections are very common. The course of disease ranges from mild to severe and sometimes even life-threatening infections. Optimal therapy is based on rapid abscess incision and, especially in severe cases, on adjuvant antibiotic therapy that ideally targets the culprit bacteria. In order to identify these bacteria, clinicians usually perform cultural analysis from smears of pus and aim for antibiotic susceptibility testing. In recent years, using new molecular methods, it has become possible to carry out a much more detailed analysis of the bacterial colonization of different parts of the human body by determining a microbiome. In our study, we have, for the first time, compared such a microbiome of odontogenic abscesses with cultural bacterial determination carried out in the clinical routine of a university hospital. The key finding of the study is not only that considerably more bacteria can be detected in the abscess in this way but also that easily cultivated bacteria dominate over the actual fastidious pathogenic bacteria. Thus, routine clinical culture probably only provides a distorted picture of reality and should be supplemented by molecular methods in the future.

**Abstract:**

Odontogenic abscesses are usually caused by bacteria of the oral microbiome. However, the diagnostic culture of these bacteria is often prone to errors and sometimes fails completely due to the fastidiousness of the relevant bacterial species. The question arises whether additional pathogen diagnostics using molecular methods provide additional benefits for diagnostics and therapy. Experimental 16S rRNA gene analysis with next-generation sequencing (NGS) and bioinformatics was used to identify the microbiome of the pus in patients with severe odontogenic infections and was compared to the result of standard diagnostic culture. The pus microbiome was determined in 48 hospitalized patients with a severe odontogenic abscess in addition to standard cultural pathogen detection. Cultural detection was possible in 41 (85.42%) of 48 patients, while a pus-microbiome could be determined in all cases. The microbiomes showed polymicrobial infections in 46 (95.83%) cases, while the picture of a mono-infection occurred only twice (4.17%). In most cases, a predominantly anaerobic spectrum with an abundance of bacteria was found in the pus-microbiome, while culture detected mainly *Streptococcus*, *Staphylococcus,* and *Prevotella* spp. The determination of the microbiome of odontogenic abscesses clearly shows a higher number of bacteria and a significantly higher proportion of anaerobes than classical cultural methods. The 16S rRNA gene analysis detects considerably more bacteria than conventional cultural methods, even in culture-negative samples. Molecular methods should be implemented as standards in medical microbiology diagnostics, particularly for the detection of polymicrobial infections with a predominance of anaerobic bacteria.

## 1. Introduction

Odontogenic infections are among the most common inflammatory diseases in the head and neck region [1,2]. Frequently, these infections lead to abscesses, which can usually be treated by incision and drainage and, if necessary, by concomitant antibiotic therapy [3]. The spectrum of these infections ranges from minor abscesses in the oral cavity, which can be easily treated in the dental practice, to extensive and sometimes life-threatening abscesses of the entire head and neck region, which require hospital treatment under general anesthesia and can even lead to death [4,5]. In addition to incision and drainage of the abscess, adjuvant antibiotic therapy is becoming increasingly important in the case of extensive and advanced disease [6].

Odontogenic infections are usually polymicrobial endogenous infections that are generally well amenable to empirical antibiotic therapy. However, as in other fields of medicine, resistance to antibiotics has been increasingly observed in the recent past, which can occasionally complicate such empirical antibiotic therapy [7,8]. Particularly in extensive infections, cultural pathogen diagnostics are performed to enable targeted antibiotic treatment according to an antibiotic susceptibility profile, especially if the initial therapeutic interventions are not optimally effective [7].

Numerous authors have shown that odontogenic infections are mainly caused by anaerobic bacteria [7,9,10]. However, these are very difficult to detect culturally [7]. Therefore, high demands must be placed on pre-analytic considerations such as sampling, the transport medium, and the entire time sequence until pathogen diagnostics in the laboratory. Otherwise, incomplete or even incorrect bacterial determination has to be expected. Thus, culture-negative and possibly sterile abscesses are frequently reported, in which no bacteria are culturally detectable despite an extensive clinical disease appearance [11].

Using modern molecular biological methods, it became possible to show that many pathogens are usually involved in odontogenic infections, even more than could previously be detected with a classical cultivation approach [12,13]. In particular, using such methods in combination with next-generation sequencing procedures has enabled increasingly comprehensive diagnostics, allowing the detection of microorganisms that have not yet been cultivated [14]. The determination of bacterial composition by sequencing amplified 16S rRNA genes is also called the determination of a microbiome [15], which corresponds to the determination of a pus-microbiome in the case of an odontogenic abscess. Since 16S rRNA gene analysis cannot detect any fungi or viruses, the term bacteriome would also be justified. However, molecular amplicon sequencing methods do not allow the prediction of antibiotic susceptibility, as is possible using classical cultural pathogen detection [16]. Concerning the clinical routine of pathogen diagnostics in odontogenic abscesses, the question arises about the diagnostic value of the determination of a pus-microbiome. This study aims to give answers to the following clinical questions:

1. Which bacteria can be detected that may not entirely or partially be detected by cultural methods alone?
2. Is it possible to improve the accuracy and completeness of the established cultural methods with such additional diagnostics?
3. Do biomolecular pathogen diagnostics offer advantages in comparison to classical culture?

## 2. Materials and Methods

All patients hospitalized and treated for odontogenic abscesses between October 2016 and March 2017 in the Department of Oral and Maxillofacial Surgery of the University Hospital Giessen were included in the study until a maximum possible number of 50 samples was reached. This number corresponded exactly to the capacity that could be made available in terms of finance and personnel for this exploratory study. The patients gave their written consent to participate in the study prior to surgery, and the project was authorized by the local Ethics Committee of the Medical Faculty of the Justus-Liebig-University Giessen (Vote 191/16). Part of the data has already been used for a comparison between oral and pus microbiomes in odontogenic abscesses [17].

Clinical therapy was utterly independent of the study. Swabs (wrapped fiber swabs with gel-based Amies medium) were taken during the abscess incision from drained pus and used for routine microbiological examination by culturing and antimicrobial susceptibility testing (aerobic and anaerobic culture). Additionally, pus was obtained from the incised abscess and frozen at −80° degrees Celsius for later molecular biological evaluation. The abscesses were drained continuously using easy-flow drains, and therapy was monitored clinically and by routine laboratory examination until hospital discharge.

Aerobic and anaerobic culture was performed using Columbia blood agar, chocolate blood agar, McConkey agar, Sabouard glucose agar, Schaedler/Schaedler KV agar (biplate), and Thioglycollate broth. The media were incubated at 37 °C and read after 24 and 48 h except for Schaedler/Schaedler KV agar plates, which were incubated for 48 h before reading. An additional Schaedler/Schaedler KV agar plate remained in incubation for 5 days before reading. Bacteria were identified using a MALDI-TOF system (Vitek MS, Biomérieux, Nürtingen, Germany). Antimicrobial susceptibility testing was performed on an automated system (Vitek 2, Biomérieux, Nürtingen, Germany) or by disk diffusion or gradient strip testing according to EUCAST standards. Breakpoint interpretation was also carried out according to EUCAST standards.

Irrespective of the clinical therapy and culture results thereby obtained, the microbiome of the pus was determined from the frozen samples about three months later using 16S rRNA gene analysis [18,19,20]. 16S rRNA amplicon sequencing works like a bacterial fingerprint if the base sequence can be matched with a ribosomal database. It is particularly useful in identifying unusual bacteria that are difficult to identify by conventional methods, providing genus identification in >90% of cases [21]. Therefore, nucleic acid was first extracted from the initially frozen pus samples, as previously described [17]. Then, the V4 region of the 16S rRNA gene was amplified by polymerase chain reaction using primers in the conserved flanking areas with adapters [17]. The resulting amplicons of an approximate length of 350 to 370 bps were processed for next-generation sequencing using the Illumina MiSeq system, as described by the vendor (Illumina, San Diego, CA, USA).

For bioinformatic analysis, paired-end sequence reads were joined and primer sequences were removed, as previously described [22]. Sequence reads varied between 28,698 and 751,847 per sample. Reads with ambiguous base calls or with homopolymers longer than eight nucleotides were removed and duplicates were merged and aligned against the SILVA bases’ bacterial reference alignment [23]. Applying Mothur implementation of the UCHIME algorithm, chimeric reads were removed, taxonomy was assigned, and non-bacterial reads were removed from the analysis. Operational taxonomic units (OTU) were generated, and taxonomy was reassigned using Mothur. In preparation for the analysis, an OTU table in biom format was created.

Statistical analysis was carried out with Microsoft Excel (Redmond, WA, USA) and the statistical software R-4.0.4 (R Core Team, 2021, Vienna, Austria). For calculation of frequencies, we considered a phylotype to be abundant if it contributed to at least 0.01% of the microbiome [17]. Lilliefors (Kolmogorov–Smirnov) test (R package: nortest) was applied to test normality distribution. Culture results were presented in a pie chart. To describe the composition of the microbiomes, a pie chart with the medians of the relative frequencies of the reads and a heatmap was created. The heatmap was also based on the relative frequencies of the reads and performed with hierarchical clustering and dendrogram (complete linkage with Euclidean distance; sum of rows > 20%). To compare the culture results with the microbiomes, Fisher’s exact test and a tabular comparison of each culture result and the corresponding microbiome were performed.

## 3. Results

**Figure 1:**
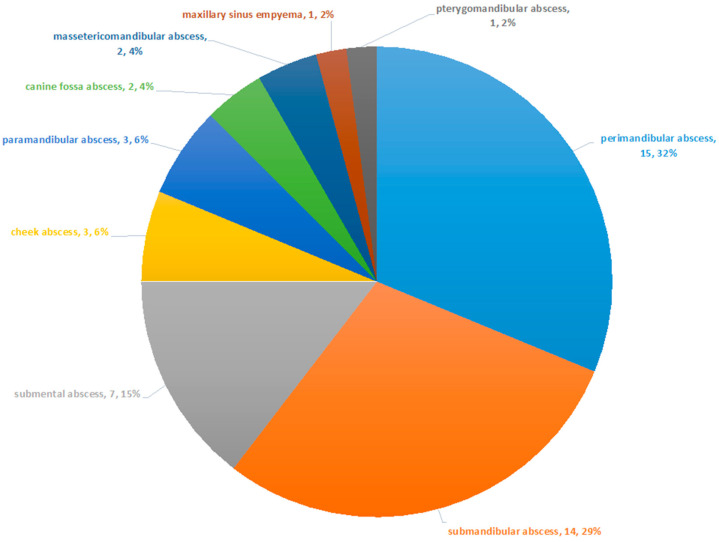
Frequency of observed abscesses. The most common abscesses were the perimandibular and the submandibular abscess.

In the observation period from October 2016 to March 2017, a total of 50 hospitalized patients with a severe odontogenic abscess were treated with incision and drainage at the University Hospital. Two patients had to be excluded because of missing swab samples, allowing the evaluation of 48 patients. In total, 16 patients (33.3%) were female, and 32 patients (66.7%) were male. The mean age was 47.79 years (standard deviation: 19.55 years). Lilliefors normality test showed that the distribution of the patient’s age did not differ significantly from a normal distribution (*p* = 0.3321). The most observed abscesses were the perimandibular abscess with 15 occurrences (32%) and the submandibular abscess with 14 (29%) occurrences (Figure 1).

**Figure 2:**
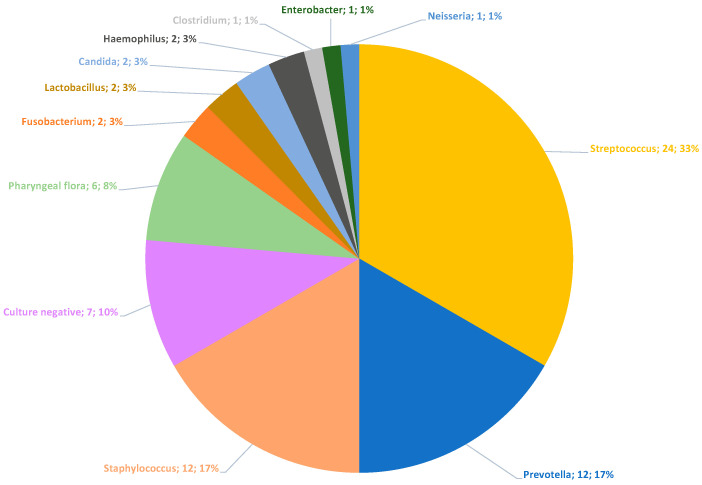
Culture results: The figure shows the absolute and relative frequency of the specifications given by the microbiological lab.

**Figure 3:**
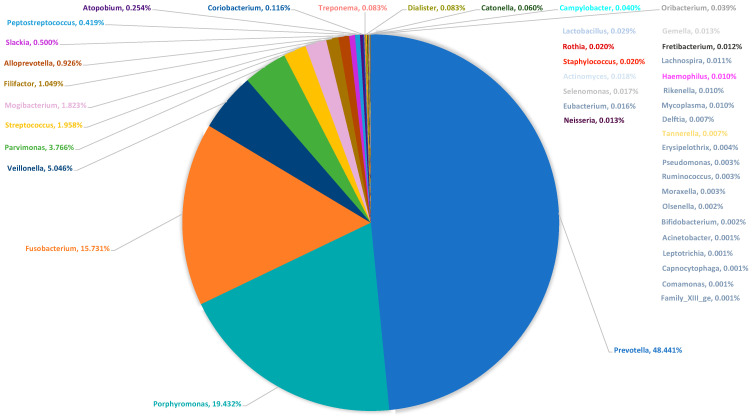
Median microbiome of the pus samples (*n* = 48). The pie chart shows the median of the relative frequencies. The median was used because a normal distribution of the relative frequencies of the genera was not given.

**Figure 4:**
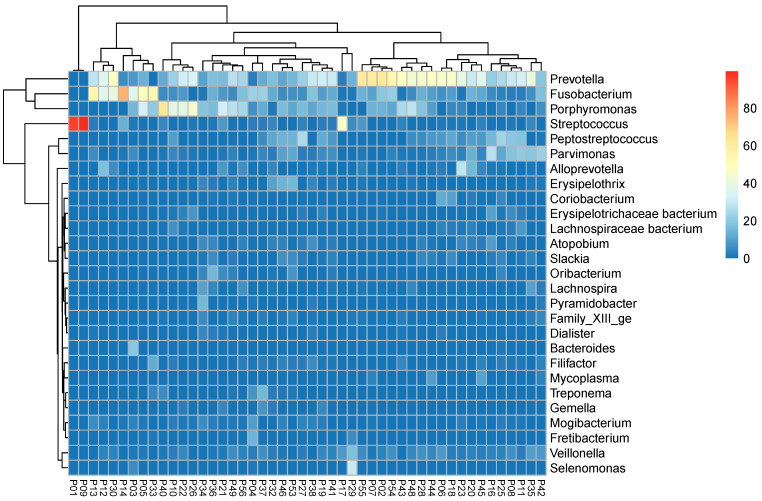
Heatmap of all (*n* = 48) pus samples. Colors show the relative frequency of the reads of the most frequent bacterial genera (sum of rows >20%). Hierarchical clustering with a dendrogram (complete linkage with Euclidean distance).

**Figure 5:**
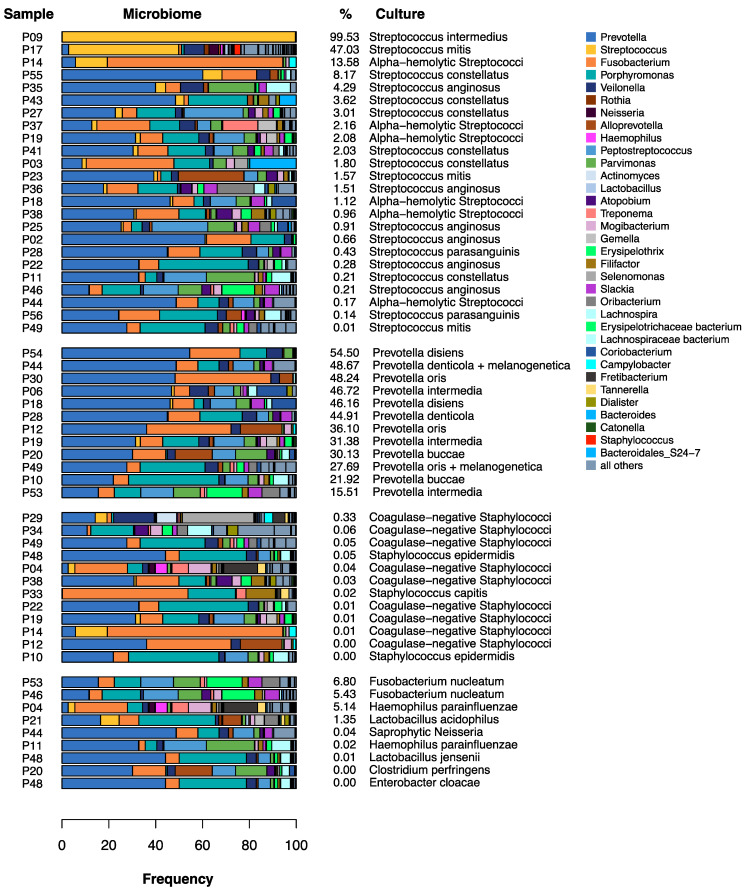
Culture results compared to the corresponding microbiomes. Every culturally detected bacterial species was compared directly with the corresponding microbiome. The percentage number indicates how many reads fall on the genus of the cultured species. The frequently cultured genera *Streptococcus* (yellow) and *Staphylococcus* (red) are only seen very sporadically in the microbiomes.

The information of the microbiological findings was given either by the correct identification of a bacterial species or by information such as “culture-negative” or “pharyngeal flora” (which represents a mixture of commonly colonizing species of the oro-pharyngeal space). In this context, each statement made by the microbiological lab was evaluated as a specification. In most cases, a specification corresponded to a cultural reference. In 48 cases, this resulted in a total of 72 specifications (Figure 2). In 23 cases (47.92%), one specification was submitted; in 12 cases (25.0%), two specifications were given; and in 6 cases (12.5%), a maximum of three specifications was reported. In 7 of 48 cases (14.58%), no microorganisms could be cultured (culture-negative). Thus, cultural bacterial detection was successful in 41 of 48 patients (85.42%). Figure 2 shows the type of specifications and their overall frequency. For later comparison with the pus-microbiome (Figure 3 and Figure 4), the specifications were summarized at the genus level. In addition, Figure 5 shows the culture results down to the species level for all samples.

*Streptococci* were cultivated most frequently, with 33.3% of the specifications. Figure 5 shows that these were exclusively *alpha-hemolytic Streptococci*. *Prevotelles* and *Staphylococci* were found, each with 16.6% of the specifications (Figure 2). Figure 5 shows that all recovered *Staphylococci* were coagulase-negative.

16S rRNA gene analysis revealed the microbiome of the relieved pus. As described by other authors [12], an abundance of bacterial genera was found in contrast to culture. We considered a phylotype to be abundant if it contributed to at least 0.01% of the microbiome. Using this threshold, a mean of 31.42 (±12.30) was found in the pus, and the Lilliefors normality test indicated a normal distribution of the number of bacterial genera in the pus-microbiome (*p* = 0.3138). Figure 3 shows the median microbiome of the pus, in which the median of the relative abundances was summed up and extrapolated to 100%.

In this way, a direct comparison between the results of the culture and the 16S rRNA gene analysis was possible. Figure 2 and Figure 3 show that the detection of easily cultivable *Streptococci* and *Staphylococci* obviously dominates culture results in contrast to the molecular microbiome determination. Here, a strong predominance of typically anaerobic genera such as *Prevotella* (blue color), *Fusobacterium* (orange color), and *Porphyromonas* (turquoise color) is shown. It is also evident that a maximum of three different species was detected in culture, while, on average, more than 30 bacterial genera could be demonstrated in the microbiome. Thus, the routine clinical culture obviously only represented a small part of the potentially present pus-microbiome, in which easily cultivable bacteria such as *Streptococcus* and *Staphylococcus* were overrepresented compared to fastidious anaerobic bacteria. Only in 2 of the 48 cases (4.17%) was a clear predominance of *Streptococcus* observed, while the picture of a polymicrobial infection was seen in all other samples (95.83%).

Figure 4 shows a heatmap regarding the composition of the pus-microbiomes of the most common bacterial genera. Even here, it is evident that *Streptococcus* only occurs twice as an actual “culprit pathogen “(samples P1 and P9) and that *Staphylococcus* is almost totally absent.

Among the specifications given by the microbiological lab, a total of 56 bacterial species could be identified. The genera of these bacteria were tested for differences in their frequency of occurrence using Fisher’s exact test. For this purpose, an occurrence in the microbiome was evaluated as long as the relative frequency of the reads was at least 1%. Table 1 shows that culture and microbiome differ significantly according to their results (*p* < 0.001).

In Figure 5, the culture results are directly compared to the corresponding microbiomes. The colors show that only in cases of cultural detection of *Prevotella,* there was also a significant proportion of *Prevotella* (blue) in the corresponding microbiome. If *Streptococcus* was detected, the microbiome only showed a more substantial proportion of *Streptococcus* (yellow) in some cases, and *Staphylococcus* (red) was almost not present at all in the microbiomes, although *Staphylococcus* was frequently cultured.

For some of the cultivated bacteria, the Institute of Medical Microbiology also provided antimicrobial susceptibility testing (EUCAST standards) in addition to the culture results. The resistances of the three most frequently cultivated bacterial genera (*Streptococcus*, *Staphylococcus*, and *Prevotella*) are given in Table 2. For the genus *Prevotella*, which showed by far the highest frequency in the median microbiome, resistance to penicillin was shown in almost 79% of cases and to clindamycin in nearly 43% of cases. Genus *Streptococcus* still showed good susceptibility overall. In particular, resistance to the frequently used combination of ampicillin and sulbactam could only be determined in one case.

## 4. Discussion

Odontogenic abscesses are the most common inflammatory diseases in the oral and maxillofacial region [2,24]. Such infections are endogenous and can be prevented by adequate pre-emptive care [25,26]. The severity ranges from mild abscesses, which can be treated on an outpatient basis in the dental office, to severe life-threatening conditions requiring hospital or even intensive care [4,5]. If antibiotic therapy is needed, it is usually performed immediately after abscess incision as empirical therapy, often using ampicillin with sulbactam or clindamycin in the case of penicillin allergy [27].

### 4.1. Does Culture Provide the Identification of the “Culprit Pathogen”?

It is often recommended to take a swab as part of an abscess incision and to aim for cultural bacterial detection with the preparation of an antibiogram. With the knowledge of a “culprit pathogen”, specific antibiotic therapy can then be administered instead of a calculated one, tailored precisely to this pathogen [7]. However, what value does an antibiogram have if instead of one culprit bacterium, there are many potentially culprit bacteria or even a culprit community, and what if many of these bacteria cannot be detected at all or only with particularly great effort?

Many authors have shown that odontogenic abscesses are usually polymicrobial [7,9,10]. While about three to eight bacterial species can be identified by cultural methods, up to 114 bacteria can be identified in odontogenic abscesses by more recent molecular biological techniques [12]. Thus, also in this work, a mean of 31.42 bacterial genera was determined using 16S rRNA gene analysis. As shown in Figure 4, only 2 out of 48 cases (p1 and p9) showed the picture of a real mono-infection with the genus *Streptococcus* as a “culprit pathogen”, while the remaining 46 cases showed a mixed picture, matching the polymicrobial etiology. As reported by other authors [12,13], obligate anaerobic bacteria such as *Prevotella*, *Porphyromonas,* and *Fusobacterium* predominate with regard to the relative frequencies of the reads (Figure 3 and Figure 4).

Other genera that could have played a role in the pathological community due to their relative abundance were *Veillonella*, *Parvimonas*, *Streptococcus*, *Mogibacterium,* and *Filifactor* (Figure 4). Thus, in the vast majority of cases of odontogenic abscess, an appropriate antibiogram would always need to cover a wide range of bacteria or at least identify those pathogens that have clinically relevant resistance to antibiotics. Theoretically, this could also be a key species of the pus-microbiome with a very low abundance, which secondarily enables the proliferation of more abundant species. For instance, it has been reported for *V. parvula* that the species produces menaquinones that can meet the specific nutrient requirements of *Porphyromonas* spp. and *Prevotella* spp. for this substance [28]. Viral co-infections could theoretically also have an influence on the composition of the culprit bacterial community and, thereby, on its susceptibility to antibiotics.

In this study, a scientific analysis of the microbiome of odontogenic abscesses was compared with the results of routine clinical examinations. A possible weakness of the study was that although the samples were taken at the same time from the same patient, culture and 16S rRNA gene analyses were performed from different transport containers. It was also not reasonably possible to determine the influence of a possible antibiotic pretreatment on the culture and microbiome results. Many patients have probably already received orally administered antibiotics prior to hospitalization. However, patients often provide insufficient information in this regard, and, at the same time, a vast choice of possible substances has to be considered, which makes a serious evaluation of this information difficult. On the other hand, it is widely accepted that significant infectious swellings cannot be influenced by applying oral antibiotic medication alone [4,27]. Therefore, it is most likely not of significant importance what kind of antimicrobial oral medication was administered prior to hospital admission and surgical treatment. With regard to aspects of quality assurance, this disadvantage may also be an advantage, as the cultural analysis corresponded to the real conditions of clinical routine. Usually, the surgeon does not have better microbiological diagnostics at his disposal to make therapeutic decisions, and, against this background, additional molecular biological investigations seem to be of particular relevance.

The most frequently identified bacterial genera in the cultural analysis were *Streptococcus*, *Staphylococcus,* and *Prevotella* (Figure 2). Table 1 and Figure 5 show that only the genera *Streptococcus* and *Prevotella* were present to any significant extent in both the microbiome and the culture. The genera *Porphyromonas*, *Parvimonas*, *Veillonella*, *Mogibacterium,* and *Filifactor*, on the other hand, were culturally not detected at all, and *Fusobacterium* was only identified in two cases, even though *Porphyromonas* and *Fusobacterium*, in particular, accounted for a considerable proportion of the median microbiome, with about one-third (Figure 3), and *Fusobacterium* and *Parvimonas* have been associated with acute apical odontogenic infections by other authors [13]. Thus, cultural analysis performed in routine clinical practice was obviously unable to correctly determine the “culprit pathogen “or “culprit community” of the abscesses.

In contrast, *Staphylococci* were almost not detected in the microbiome, but they were identified in culture as frequently as the genus *Prevotella*. This suggests a contamination of the swabs by the physiological skin flora during swab collection or the proliferation of *Staphylococci* during the time period from sample acquisition until the start of laboratory examination. Even if cultural analysis, if carried out with a more appropriate effort than in clinical routine, can certainly provide better results than this study, it must still be suspected that culture methods alone offer a distorted picture of the relevant microbiota composition. If *Streptococcus* and *Staphylococcus* prevail in culture according to the principle of “survival of the fittest”, the detection of the obligate anaerobic genera *Prevotella*, *Fusobacterium,* and *Porphyromonas* would hardly succeed to a sufficiently reliable degree. Figure 5 shows that if *Streptococci* were culturally detected, the relative abundance of the genus *Streptococcus* in the microbiome was only four times above 5%. This suggests that the readily culturable *Streptococci* have indeed prevailed in culture against the difficult-to-cultivate fastidious anaerobes. Another explanation could be that certain bacteria have special requirements for their culture media so that some of them still could not be cultivated at all [14].

To obtain a valid culture result and an antibiogram with regard to the anaerobes, we suggest the following recommendations:

1. Careful disinfection of the skin (or mucosa) at the incision site: in this way, contamination by *Staphylococci* from the skin can be prevented [29].
2. Swabbing the depth of the abscess cavity instead of the flowing pus on the skin surface of the incision site: Pus itself represents a hostile medium for bacteria. Swabbing the living tissue at the depth of the abscess may increase the likelihood of obtaining living anaerobes for culturing while decreasing the likelihood of contamination by skin flora.
3. Selection of a suitable transport medium: Liquid-based media with a flocked swab can be a better alternative compared to gel-based media with traditional fiber swabs [30]. These media probably allow sufficient survival of the anaerobes and, at the same time, still enable further processing by molecular biological methods [31,32].
4. Cooling to 2–4 °C and fastest possible transport to the microbiological laboratory: The later the transport, the less likely the swabbed bacteria can be recovered in the laboratory [31,32].

### 4.2. Is Swab Collection Necessary at All?

Even if swab collection and culture are performed under optimal conditions in the case of odontogenic infections, in the end, not all bacteria of the pathological community will always be detectable [14]. The question remains open as to whether a swab should be taken at all. In the authors’ opinion, this is not necessary for simple clearly odontogenic abscesses and otherwise healthy patients, provided that rapid healing can be expected even without the use of antibiotics. Accordingly, the German guideline on odontogenic abscesses recommends that intraoperative pathogen diagnosis can be aimed for, but it is not mandatory [27]. Swabbing becomes important whenever a possible complicated course of the disease is expected or an odontogenic cause of the infection cannot be proven. Particularly in patients with immune insufficiency and systemic (poor general condition, chemotherapy, bisphosphonate therapy) or local damage (radiation therapy), it has to be taken into account that the supposedly odontogenic infection can also be caused by other causes and possibly be based on an entirely different pathogen spectrum [33]. In this case, taking a swab can help distinguish classic mono-infections, which are often caused by easily culturable bacteria, from the typical anaerobic pathogen spectrum of odontogenic infections. In particular, infections with pyogenic *Streptococci* (e.g., peritonsillar abscess), abscesses caused by *Staphylococci* (especially *S. aureus*), and infections with nonfermenting Gram-negative bacilli (NFGNB) [20] should be mentioned here, in which a possible adjuvant antibiotic therapy may need to be designed differently for the typical odontogenic abscess.

### 4.3. Does Antibiotic Resistance Have Clinical Relevance in Odontogenic Infections?

The microbiomes indicate that effective antibiotic therapy needs to target typical anaerobic bacterial genera such as *Prevotella*, *Fusobacterium,* and *Porphyromonas* on one hand and the genus *Streptococcus* on the other. The genus *Staphylococcus* has been frequently detected culturally, but due to its low relative abundance in the microbiome, it probably corresponds more to contamination from the skin and, therefore, does not primarily need to be considered when selecting an antibiotic therapy. Table 2 shows that few resistances have been documented against the commonly used and recommended combination of ampicillin and sulbactam [27]. In only one case, the genus *Streptococcus* showed resistance in this study, while the genus *Prevotella* was sensitive without exceptions. This was different when looking at clindamycin, which is frequently used, especially by dentists [16], particularly if penicillin allergy or penicillin intolerance is a concern [16,27]. Here, the genus *Streptococcus* showed resistance in 18.75% of cases and the genus *Prevotella* in 42.86%. Thus, in our study, almost half of the cultured *Prevotella* spp. were resistant to clindamycin. This seems to confirm the trend since anaerobes increasingly develop resistance to antibiotics [16,34]. A recent Belgian study reported that *Prevotella* spp. were resistant to clindamycin in only 9% of cases in the 1990s, whereas in 2012, resistance to clindamycin was observed in 31% of cases [35]. Increasing resistance to clindamycin has also been reported for the genera *Porphyromonas* and *Fusobacterium* [7,8,34]. Brook et al. reported that up to 10% of studied cases showed resistance to clindamycin [16]. In contrast, such extensive resistance has not been described against the combination of an aminopenicillin with a beta-lactamase inhibitor. Hence, it is considered the first-choice therapy for odontogenic infections [27,36].

In the light of resistance developments, a combination of metronidazole and a fluoroquinolone such as moxifloxacin may be a better choice than clindamycin alone in the case of penicillin allergy [37]. In our work, *Prevotella* spp. were shown to be 100% sensitive to metronidazole, while *Streptococci* were sensitive to levofloxacin without exception. In the 2014 study by Wybo et al., 96% of *Prevotella* spp. and 100% of *Fusobacteria* showed sensitivity to metronidazole, and the sensitivity to all anaerobic isolates overall was 92% [35]. However, metronidazole alone shows no effect against microaerophilic *Streptococci*. Thus, a combination with a fluoroquinolone is recommended for polymicrobial infections [16,27]. The fluoroquinolone moxifloxacin shows good efficacy against anaerobes in contrast to older fluoroquinolones, but it is also notable for good efficacy against *Streptococci* in particular [16,38]. Significantly fewer resistance problems should arise overall against the combination of these two antibiotics compared to the use of clindamycin alone as a mono-substance, similar to the combination of an aminopenicillin with a beta-lactamase inhibitor [36].

No resistance problems would be expected using the reserve antibiotics piperacillin/tazobactam and meropenem, as seen in Table 2. With these substances, a resistance of only one *Streptococcus* sp. to piperacillin/tazobactam could be demonstrated. As presented in many studies, both substances show excellent efficacy in polymicrobial combined aerobic–anaerobic infections, with extremely favorable sensitivities. However, in the authors’ opinion, these substances should be reserved for particularly extensive cases, namely, patients with pronounced immunosuppression and severe infections that are not clearly of odontogenic origin, because, in such cases, bacteria other than the typical odontogenic pathogenic milieu have to be expected [33].

Finally, we point out that the most essential treatment for odontogenic abscesses is incision and drainage, according to Galen’s almost 2000-year-old statement “*ubi pus, ibi evacua*” [11]. Most odontogenic abscesses can be treated in this way without any antibiotics at all, and, even in more extensive abscesses requiring hospitalization, adjuvant antibiotic therapy can often be avoided [6,39].

## 5. Conclusions

Concerning microbiological pathogen diagnostics, it can be stated that bacterial culture faces limitations based on pre-analytics, methodology, and pathogen specifications and, in many cases, probably represents only a tiny part of reality. A holistic approach, combining biomolecular pathogen diagnostics by 16S rRNA gene analysis, next-generation sequencing, and bioinformatics with cultural pathogen diagnostics should be implemented in clinical routine as soon as possible. Only in this way will it be possible to be informed of the content and origin of abscesses in the oral and maxillofacial region wholly and reliably. Molecular methods are predestined to become the gold standard in medical microbiology diagnostics, particularly for polymicrobial infections with a predominance of anaerobic bacteria.

## Figures and Tables

**Table 1:** 56 specifications of the culture analysis corresponded to a unique bacterial identification. These were compared with the corresponding identifications of the microbiome, requiring a relative abundance of at least 1%. Fisher’s exact test shows a significant difference between culture and microbiome (*p* < 0.001).

	Culture	Culture (%)	Microbiome	Microbiome (%)
*Prevotella*	12	21.43	45	39.82
*Fusobacterium*	2	3.57	25	22.12
*Streptococcus*	24	42.86	35	30.97
*Neisseria*	1	1.79	1	0.89
*Haemophilus*	2	3.57	3	2.66
*Staphylococcus*	12	21.43	2	1.77
*Clostridium*	1	1.79	0	0.00
*Lactobacillus*	2	3.57	2	1.77
	56	100	113	100

**Table 2:** Resistance to the bacterial genera cultivated in this study. For the three most frequent genera, the absolute and relative amounts of resistance were determined. The genus *Prevotella*, which showed by far the highest frequency in the microbiome, showed resistance to penicillin in 79% of cases and clindamycin in 43% of cases.

	*Streptococcus*				*Staphylococcus*				*Prevotella*			
*Penicillin*	2	/	17	11.76%					11	/	14	78.57%
*Ampicillin/Sulbactam*	1	/	14	7.14%					0	/	14	0.00%
*Clindamycin*	3	/	16	18.75%	1	/	3	33.33%	6	/	14	42.86%
*Metronidazol*	2	/	2	100.00%					0	/	14	0.00%
*Piperacillin/Tazobactam*	1	/	11	9.09%					0	/	14	0.00%
*Meropenem*	0	/	8	0.00%					0	/	13	0.00%
*Vancomycin*	0	/	16	0.00%	0	/	3	0.00%	12	/	12	100.00%
*Levofloxacin*	0	/	11	0.00%	0	/	1	0.00%	0	/	1	0.00%
*Cotrimoxacol*					0	/	3	0.00%				

## Data Availability

The datasets generated and/or analyzed during the current study are available from the corresponding author upon reasonable request.
